# Facilitated acquisition of eyeblink conditioning in those vulnerable to anxiety disorders

**DOI:** 10.3389/fnhum.2013.00348

**Published:** 2013-07-05

**Authors:** Meghan D. Caulfield, J. Devin McAuley, Richard J. Servatius

**Affiliations:** ^1^Graduate School of Biomedical Sciences, University of Medicine and Dentistry of New JerseyNewark, NJ, USA; ^2^New Jersey Medical School, Stress and Motivated Behavior Institute, University of Medicine and Dentistry of New JerseyNewark, NJ, USA; ^3^Department of Psychology, Michigan State UniversityEast Lansing, MI, USA

**Keywords:** behavioral inhibition, classical eyeblink conditioning, trait anxiety, anxiety vulnerability, temperament, anxiety

## Abstract

Behavioral inhibition (BI) increases vulnerability to develop anxiety disorders and is typified by avoidance and withdrawal from novel objects, people, and situations. The present study considered the relationship between BI and temperamental risk factors, such as trait anxiety and acquisition rate of a classically conditioned eyeblink response. One-hundred seventy-four healthy undergraduate students (mean age 20.3 years, 71.8% female) were given the State-Trait Anxiety Inventory and a battery of self-report measures of BI consisting of the Adult and Retrospective Measures of Behavioral Inhibition (AMBI/RMBI) and the Concurrent and Retrospective Self Report of Inhibition (CSRI/RSRI). Participants then underwent standard delay classical eyeblink conditioning consisting of 45 trials with a 500-ms CS overlapping and co-terminating with a 10-ms airpuff US. Individuals with higher scores on the AMBI and Trait Anxiety Inventory, but not the other measures, showed faster acquisition of a conditioned eyeblink response than individuals with lower scores. Results support a relationship between facilitated acquisition of inter-stimulus relationships and risk for anxiety, and suggest that some measures assessing anxiety vulnerability better capture this relationship than others.

## Facilitated acquisition of eyeblink conditioning in those vulnerable to anxiety disorders

One quarter of the US population is estimated to develop an anxiety disorder at some time in their lives (Kessler et al., 2005a,b). Another way to look at this statistic is that 75% of Americans do not develop clinical anxiety, raising the question of what is it about an individual that makes them more or less likely to develop an anxiety disorder? So far, it appears that there is no single factor that increases one's risk for developing an anxiety disorder. Rather, anxiety disorders are best represented by a combination of pre-existing factors that reflect enhanced vulnerability to anxiety, following the experience of stressors in the environment. A stress-diathesis model for the development of anxiety disorders emphasizes changes in stress reactivity following the convergence of a variety of factors such as genetics, biology, sex, personality, and prior experience (Mineka and Zinbarg, 2006). While all of these factors require associations between the environment and stressors, present diathesis models of anxiety vulnerability do not take into account individual differences in learning.

Behaviorally inhibited temperament is a personality risk factor linked to increased likelihood to develop anxiety disorders (Biederman et al., 1990, 2001; Hirshfeld et al., 1992; Schwartz et al., 1999). Behaviorally inhibited individuals demonstrate similar behavioral and physiological profiles as seen in clinical anxiety including withdrawal, apprehension, slow latency to approach unfamiliar people or objects (Kagan et al., 1988; Kagan, 1997), altered andrenocortical activity (Schmidt et al., 1997) reduced heart rate variability and increased bradycardic responses (Garcia Coll and Kagan, 1984; Kagan et al., 1987).

Avoidance is the core feature of both clinical anxiety and behavioral inhibition (BI) (Kagan et al., 1987; American Psychiatric Association, 2000; Morgan, 2006; Schofield et al., 2009). As such, understanding the role of avoidance in the development and maintenance of anxiety is essential. Avoidance is a learned response that is acquired and reinforced over time. Rather than deal with uncontrollable events, anxious individuals assert control by substituting other negative thoughts or feelings that are avoidable, providing a feeling of control and temporary relief while at the same time increasing the aversiveness of the undesired stimulus or state in the future, ensuring continued avoidant behavior (Mineka and Zinbarg, 2006). Over time, avoidant behaviors become pervasive and uncontrollable such that normal functioning becomes impossible. Because avoidance is a learned process it is possible to measure the acquisition of negative reinforcement contingencies. Individual differences in the speed of acquisition or strength of associations in avoidance may contribute to vulnerability or resiliency. Certain individuals may be more susceptible to acquire and repeatedly express avoidant behaviors, such as those who are behaviorally inhibited, leading to the development of behavioral and cognitive avoidance symptoms associated with clinical anxiety.

Multiple processes underlie avoidance acquisition and maintenance such as sensitivity to acquire inter-stimulus associations and rigidity of expression making it difficult to sift out the essential factors leading to anxiety disorders. One possibility is that increased sensitivity to cues and contingencies in the environment are learned faster in anxious individuals, resulting in better performance on avoidance tasks (Sheynin et al., 2013). Eyeblink classical conditioning provides a means to measure these associations, enabling multiple measures to be taken into account including reactivity, acquisition of the relationship between stimuli, and the rate of extinction. Rather than using operant avoidance paradigms, eyeblink conditioning is a simple and sensitive tool that benefits from an advanced understanding of the neural substrates, amenability for cross species comparisons, control over the stimulus parameters and measurability of multiple aspects of the response. The neutral substrates underlying eyeblink conditioning has been documented at length, with converging agreement that the cerebellum is both necessary and sufficient to acquire delay-type eyeblink conditioning (Thompson, 1976; McCormick et al., 1981; Swain and Thompson, 1993; Thompson and Kim, 1996; Grillon and Hill, 2003). Eyeblink conditioning is a measure of associative learning that utilizes a simple reflex pathway. In delay-type eyeblink conditioning, a tone conditioned stimulus (CS) precedes and co-terminates with a corneal airpuff unconditional stimulus (US) that elicits an unconditional response (UR). Over repeated pairings, the CS induces a conditioned response (CR) that precedes and modifies the US.

Differences in acquisition of conditioned eyeblink responses has been demonstrated in individuals demonstrating anxiousness and avoidant behaviors including anxiety (Tracy et al., 1999; Ayers and White, 2003; Burriss et al., 2007; Holloway et al., 2012) and BI (Myers et al., 2011). Spence and colleagues (Farber and Spence, 1953; Spence and Beecroft, 1954) initiated research on the relationship between anxiousness in healthy individuals and associative learning. Using the Manifest Anxiety Scale [MAS:(Taylor, 1953)], they separated healthy college-aged individuals into high and low anxious groups and then compared acquisition in eyeblink classical conditioning. In a series of studies, Spence and others found that those who scored high on the MAS demonstrated more conditioned responses (CRs) than those with low scores (Farber and Spence, 1953; Spence and Beecroft, 1954). Recently, using a similar scale of Trait Anxiety (Spielberger et al., 1983). Holloway et al. (2012) demonstrated facilitated acquisition as well as proactive interference in Trait anxiety with pre-exposures of the US attenuating learning to a greater degree in high Trait anxious individuals, suggesting those vulnerable to anxiety interpret stimuli in their environment differently. Recently, Myers et al. (2011) demonstrated facilitated delay eyeblink acquisition in veterans not reporting current severe post traumatic stress symptoms with high scores on the Retrospective Measure of Behavioral Inhibition (Gladstone and Parker, 2005) compared to low scoring individuals, indicating a relationship between behaviorally inhibited temperament and associative learning.

Parallels are evident between rat models of anxiety vulnerable temperament and humans with self-reported inhibited temperament, suggesting a common neural substrate. Similar to the behaviorally inhibited personality profile, the Wistar-Kyoto rat (WKY) demonstrates inherent anxiousness, vulnerability to stress, and avoidant behaviors (Paré, 1989a,b, 1993, 1994; Redei et al., 1994; Rittenhouse et al., 2002; Servatius et al., 2008; McAuley et al., 2009; Beck et al., 2010). WKY male rats acquire eyeblink conditioning significantly faster than outbred Sprague-Dawley rats, with greater asymptotic performance and resistance to extinction (Ricart et al., 2011).

Considering the close relationship between associative learning of cues as predictors of aversive events, enhanced classical conditioning would also be reflected in sensitivity to acquire avoidance responses. Presently, only the Myers et al. (2011) study assessed this relationship in terms of behavioral inhibition. While the veterans used were considered healthy in that they did not demonstrate post traumatic stress symptoms, it remains that the experiences of a veteran are likely very different from that of civilians. Therefore, it is important to understand how BI relates to associative learning in other healthy populations.

The current study assessed the relationship between BI and acquisition in delay eyeblink classical conditioning. To approach anxiety disorders from a vulnerability perspective we chose to use a healthy sample of college-aged individuals that minimizes present and past psychopathologies. It is important to note that while we utilized measures of anxiety vulnerability in this study, we did not conduct a structured clinical interview. Therefore, it is possible that some participants may suffer from undiagnosed anxiety disorders.

It is presently unclear which measures are effective in differentiating eyeblink acquisition of healthy individuals. Therefore, multiple measures of behavioral inhibition, the Adult Measure of Behavioral Inhibition (AMBI), the Retrospective Measure of Behavioral Inhibition (RMBI), the Concurrent Self Report of Inhibition (CSRI), and the Retrospective Self Report of Inhibition (RSRI) were used. Additionally, instead of using the MAS, which was designed specifically to separate individuals in experimental studies, we chose to use the State-Trait Anxiety Inventory (STAI-Y), which is similar in its approach but benefits from extensive validation and widespread use. Furthermore, assessing BI in addition to trait anxiety allowed evaluation of facilitated associative learning in terms of specific constructs, such as behavioral inhibition, or a general over-arching principal, such as anxiousness.

We assessed the effectiveness of these measures in separating eyeblink acquisition (as determined by the number of CRs) in high and low scoring individuals. Following eyeblink acquisition, participants received a series of CS-alone trials allowing assessment of the relationship between extinction and high and low scorers on each measurement. We hypothesized that high scoring individuals would acquire delay eyeblink conditioning faster than low scorers. Specifically, given the relationship between anxiety, avoidance, and associative learning we expected the AMBI/RMBI, which emphasizes avoidant behaviors to be the best at differentiating learning. Furthermore, given that anxiety vulnerability is a stable, long-term risk factor, we expected that STAI-Trait would differentiate learning, but not STAI-State, which is a measure of transient, temporary anxious feelings in the present.

## Methods

### Participants

One-hundred seventy-four students (*n* = 125 female, *n* = 49 male), ages 18–40 years (*M* = 20.3, *SD* = 2.8), from a large Midwestern university participated in return for partial credit in an undergraduate psychology course. All study materials were reviewed and approved by internal review and informed consent was obtained from all participants prior to any experimental procedures.

### Self-report measures

Participants completed a battery of self-report questionnaires prior to undergoing eyeblink conditioning. Participants were given the Adult and Retrospective Measure of Behavioral Inhibition (Gladstone and Parker, 2005), the Concurrent and Retrospective Measures of Behavioral Inhibition (Reznick et al., 1992) and the Spielberger State/Trait Anxiety Inventory (Spielberger et al., 1983).

The Adult Measure of Behavioral Inhibition is a 16-item self-report measure that assesses the presence of inhibition or avoidance in response to new stimuli or social situations. Items ask questions such as “Do you tend to withdraw and retreat from those around you?”, and “Do you tend to introduce yourself to new people?” to assess four underlying constructs of fearful inhibition, risk avoidance, non-approach and low sociability. Participants are asked to respond to questions on a three-point scale and indicate no/hardly ever (“0”), some of the time (“1”), or yes/most of the time (“2”). Total scores can range from 0 to 32. Similarly, the Retrospective Measure of Behavioral Inhibition is an 18-item self-report measure on the same scale of 0–2 that assesses childhood memories (during elementary school) of responding in unfamiliar situations. Total scores can range from 0 to 36. The scales demonstrate reliability with no differences in test-retest scores, and significant (*p* < 0.001) discriminant validity in separating anxiety, depression and control groups (Gladstone and Parker, 2005). Our sample demonstrated high internal consistency with Cronbach's alpha of 0.78 for AMBI and 0.86 for RMBI.

The Concurrent and Retrospective Self-Reports of Inhibition are similar to the AMBI/RMBI in that it measures behaviors consistent with BI especially in regards to withdrawal in social situations. The CSRI/RSRI is broader in its approach and utilizes a more direct method of questioning. Questions are answered on a 5-point scale with answers specific to the question wording (e.g., ranging from “0–4 days” to “more than 20 days” or from “never” to “very often”) but always going from least to most inhibited. The CSRI asks 31 self-report questions on the 5-point scale reflecting four aspects of BI including fears, behaviors that reflect fear, behaviors that express assertiveness and experiencing anxiety. Total scores on the CSRI can range from 31 to 155. Similar to the RMBI, the RSRI asks participants 30 self-report questions on the 5-point scale with total scores ranging from 30 to 150 about childhood experiences relating to the construct of BI as demonstrated by two factors of school/social (“during recess, did you play with the main group of children?”) and fear/illness (“How often did you have nightmares?”). Some questions did not load on any specific factor but are still part of the measure (Reznick et al., 1992). Both measures demonstrated high internal consistency with Cronbach's alpha of 0.82 for CSRI and 0.83 for RSRI.

The Spielberger State/Trait Anxiety Inventory is a 40-item self-report questionnaire with responses ranging from 1 (“almost never”) to 4 (“almost always”) with total scores ranging from 40 to 160. The STAI is separated into two parts, State and Trait anxiety, each consisting of 20 questions: State Anxiety is assumed to change with mood and emotion and asks questions about the current emotional state of the participant such as “I am tense” and “I feel at ease”. Trait Anxiety is a relatively stable personality characteristic and asks questions about general feelings and behaviors such as “I feel nervous and restless” and “I feel satisfied with myself” (Spielberger et al., 1983). Both measures demonstrated high internal consistency with Cronbach's alpha of 0.93 for STAI-State and 0.88 for STAI-Trait.

### Eyeblink conditioning

Eyeblink conditioning apparatus and procedures were previously described (Beck et al., 2008). Briefly, participants wore a customized David Clark aviation headset (Worcester, MA) from which auditory (tone) stimuli produced by signal generators (LabVIEW, National Instruments, Austin, TX) and a digital to analog converter (PCI-604E, National Instruments, Austin TX) were delivered. Sound levels were verified and checked for consistency with a Realistic sound meter (Radio Shack). The conditioned stimulus was an 82 dB 1200 Hz pure tone 500 ms in length. The headphones were also fitted with a boom placed 1 cm from the cornea that delivered a 5 psi airpuff US via sylastic tubing connected to a regulator and released by a computer controlled solenoid valve (Clipper Instruments, Cincinnati, OH). To record eyeblink responses, participants are fitted with three silver/silver chloride electromyography (EMG) electrodes covered in conductive gel. Two EMG electrodes are placed above and below the right eye and the third is placed on the neck as the ground electrode. The signal is passed to an isolated physiological amplifier (UFI, Morro Bay, CA) and band-pass filtered for low 10 Hz and high 0.1 Hz frequencies and amplified by 1000. The signal was sampled at 200 Hz by an analog to digital board (PCI-604E, National Instruments, Austin TX). Each session lasted approximately 40 min, during which participants watched a silent move (Toy Story) to reduce boredom and help maintain a forward-facing gaze.

### Procedure

All participants received the same battery of questionnaires (AMBI/RMBI, CSRI/RSRI and STAI State and Trait) followed by the delay-type classical conditioning. Following consent and the completion of questionnaires, subjects were fitted with EMG electrodes, the signal quality was checked and conditioning began. Initially, each participant was exposed to three US alone stimuli to establish appropriate responses to the airpuff and measure the UR prior to conditioning. Participants were conditioned with a delay procedure consisting of 45 CS-US paired trials (500-ms, 83-dB 800 Hz pure tone CS co-terminating with a 50-ms airpuff US) and 15 CS-alone trials consisting only of the 500-ms pure tone. Trials were separated by an inter-trial interval ranging from 25 to 37 s (*M* = 30 s). The duration of the entire experimental session was 1 h with eyeblink conditioning lasting approximately 40 min.

### Eyeblink data processing

For all sessions, eyelid EMG recordings were evaluated for each participant on a trial-by-trial basis. Sessions with excessive signal noise (loss of more than 10% of trials) or that demonstrated a lack of a UR were discarded and not used for further analysis. To be recorded as an eyeblink the smoothed signal must change by more than the mean activity plus 4 times the standard deviation in a 125-ms comparator window. Responses meeting this criterion and occurring within 200 ms of CS onset are scored as an α-response or orienting response, those between 200 ms after CS onset but prior to US onset are considered a CR and those occurring in response to the US are considered an UR (Beck et al., 2008).

### Data analysis

For eyeblink conditioning, the dependent measure was percent CRs within a block of trials. Repeated-measures ANOVA with within-subjects factor of block and between-subjects factors of high and low scorers based on a median split of the collected sample on AMBI/RMBI, CSRI/RSRI, and State/Trait. Nine blocks consisting of five trials each was used to assess acquisition and three blocks of five trials was used to assess extinction.

## Results

### Self-report measures of anxiety vulnerability

Mean scores for all of the self-report measures with standard deviations are shown in Table 1 separated by sex.

**Table 1:** **Descriptive summary of scores on self-report scales**.

**Survey**	**Male (*n* = 49)**	**Female (*n* = 125)**
	**Mean raw score (*SD*)**	**Mean raw score (*SD*)**
AMBI	12.9 (4.5)	12.6 (5.0)
RMBI	12.9 (6.9)	12.2 (6.9)
CSRI	69.1 (12.0)	72.1 (12.0)
RSRI	62.5 (12.7)	62.7 (14.2)
TRAIT	40.2 (8.9)	38.2 (9.0)
STATE	33.6 (9.0)	35.4 (12.1)

AMBI, Adult Measure of Behavioral Inhibition; RMBI, Retrospective Measure of Behavioral Inhibition; CSRI, Concurrent Self Report of Inhibition; RSRI, Retrospective Self Report of Inhibition. TRAIT, Trait Anxiety Inventory; STATE, State Anxiety Inventory.

A point-biserial correlation demonstrated no relationship between sex and any of the self-report measures (all *p*'s > 0.299). Correlations between survey measures are shown in Table 2; statistical significance was determined using Bonferroni corrected *p* value of 0.007. Correlations between survey measures were all positive. Notably, both adult measures of BI (AMBI and CSRI) and childhood measures (RMBI and RSRI) are more strongly correlated with each other than they are with the adult measures. Additionally, the STAI-Trait was reliably correlated with all measures of BI, especially the adult measures, while the STAI-State did not correlate with the AMBI or RMBI.

**Table 2:** **Relationship of self-report measures of anxiety vulnerability (*N* = 174)**.

**Measure**	**AMBI**	**RMBI**	**CSRI**	**RSRI**	**TRAIT**	**STATE**
AMBI	−					
RMBI	0.306^**^	−				
CSRI	0.615^**^	0.411^**^	−			
RSRI	0.274^**^	0.602^**^	0.507^**^	−		
TRAIT	0.246^**^	0.179	0.443^**^	0.259^**^	−	
STATE	0.138	0.135	0.373^**^	0.250^**^	0.349^**^	−

** Denotes significant correlations, p < 0.001.

### Eyeblink conditioning

Analysis of eyeblink conditioning data was completed for 117 participants; data from the remaining 57 were unusable due to poor signal quality, inability to stay alert throughout the 40 min session, or failure to exhibit the unconditioned response^1^. The distribution of male and female participants included in eyeblink analysis did not differ from the distribution of male and female participants who had to be excluded, *X*^2^(1, *N* = 174) = 1.201, *p* = 2.73. Included and excluded participants also did not differ in their survey scores (all *p*'s > 0.08 using independent samples *t*-tests). For the 117 participants included in the eyeblink analysis the average age was 19.9 years (SD = 1.7, range = 18–26 years) with 36 males and 81 females (69.2% female) and 14.0 years of education (*SD* = 1.4, range = 11–17 years). A repeated measures ANOVA of percent CRs revealed a significant main effect of training block, *F*_(8, 928)_ = 5.879, *MSE* = 0.05, *p* < 0.001, with visual inspection of the learning curves showing increasing acquisition of the CR throughout the training period. A repeated measures ANOVA to assess extinction over the three blocks revealed a significant effect, *F*_(2, 232)_ = 8.125, *MSE* = 0.05, *p* < 0.001, with fewer CRs in later extinction blocks.

### Self-report measures and eyeblink acquisition

For subsequent analyses, we compared the ability of the different self-report measures to differentiate fast and slow learners by first performing a median split on each measure to create a high scoring group and a low scoring group^2^ and then comparing acquisition for the two groups. We chose this approach for the following reasons: First, there are no published cutoffs defining those at risk for anxiety for the AMBI/RMBI, CSRI/RSRI, or STAI and this method afforded a conservative approach that would facilitate comparisons across surveys easier. Second, the approach of using a median split is the same approach that was used to group individuals during discriminability assessments of these scales during their validation. Average scores for the high and low scoring groups for each survey are shown in Table 3.

**Table 3:** **Summary of eyeblink conditioning groups made for comparison of high and low scores on AMBI, RMBI, CSRI, RSRI, STAI-Trait and STAI-State (*n* = 117)**.

**Survey**	**Median**	**High scorers**			**Low scorers**		
		***n***	**Mean raw score (*SD*)**	**Mean percent score (*SD*)**	***n***	**Mean raw score (*SD*)**	**Mean percent score (*SD*)**
AMBI	11.0	53	16.7 (4.1)	52.1 (12.7)	64	8.8 (2.0)	27.5 (6.2)
RMBI	11.0	54	17.9 (4.8)	43.9 (13.3)	63	6.8 (2.8)	34.2 (7.8)
CSRI	70.0	54	80.2 (8.0)	51.7 (5.1)	63	62.2 (6.1)	40.1 (3.9)
RSRI	60.0	55	71.9 (8.9)	47.9 (5.9)	62	52.5 (5.4)	35.0 (3.6)
TRAIT	37.0	56	45.3 (7.5)	56.6 (9.4)	61	31.8 (3.9)	39.7 (4.9)
STATE	33.0	55	42.5 (7.7)	53.2 (9.6)	62	26.8 (3.5)	33.4 (4.4)

Both mean raw and percent scores are presented.

SD, standard deviation; AMBI, Adult Measure of Behavioral Inhibition; RMBI, Retrospective Measure of Behavioral Inhibition; CSRI, Concurrent Self Report of Inhibition; RSRI, Retrospective Self Report of Inhibition.

Independent samples *t*-tests comparing the amplitude of the unconditioned response during trials in which only the US was presented revealed no significant differences between groups, but a correlation between UR amplitude and overall acquisition was significant, *r* = 0.343, *p* < 0.001. Consequently, UR amplitude was included as a covariate for the remainder of the analyses.

**Figure 1:**
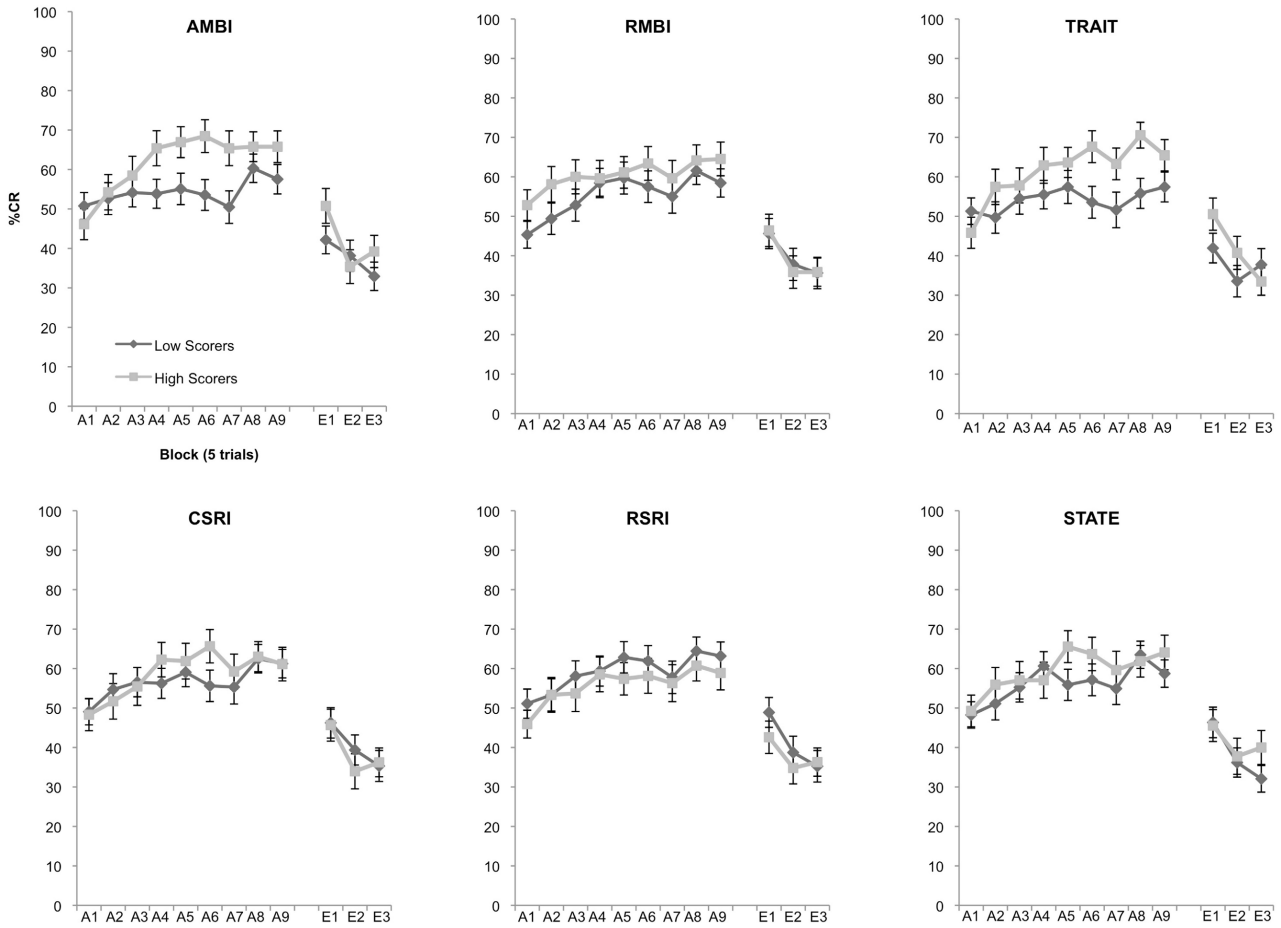
**Eyeblink conditioned responding of high and low scoring groups.** Significant differences of acquisition of the CR between high and low scoring groups were observed in the AMBI, *F*_(8, 912)_ = 2.401, *p* = 0.014, and STAI-Trait, *F*_(8, 912)_ = 3.137, *p* = 0.002. No other measures were able to significantly differentiate learning.

Using a hypothesis-driven stepwise approach we first assessed individual differences in acquisition for the AMBI and RMBI measures. Our expectation was that AMBI and RMBI would show significant differences in learning. A 2 (Group: high, low) × 9 (Block) mixed measures ANOVA revealed a significant interaction following Bonferroni correction of 0.025 between group and block, *F*_(8, 912)_ = 2.401, *p* = 0.014 for the AMBI measure but not RMBI measure, *p* = 0.725 (Figure 1). We next tested to see if the widely used BI measures of CSRI and RSRI were able to differentiate learning as well as AMBI. A 2 (Group: high, low) × 9 (Block) mixed measures ANOVA showed no significant differences in learning for groups created using the CSRI and RSRI, all *p*'s > 0.193. We then assessed learning differences in groups created using the STAI measures of State and Trait anxiety. A 2 (Group: high, low) × 9 (Block) mixed measures ANOVA demonstrated a significant interaction between group and block, *F*_(8, 912)_ = 3.137, *p* = 0.002 for the STAI-Trait measure (Figure 1) but not for STAI-State, *p* = 0.133. Finally, 2 (Group: high, low) × 3 (Block) mixed measures ANOVAs revealed no significant differences between groups in extinction, all *p*'s > 0.129.

**Figure 2:**
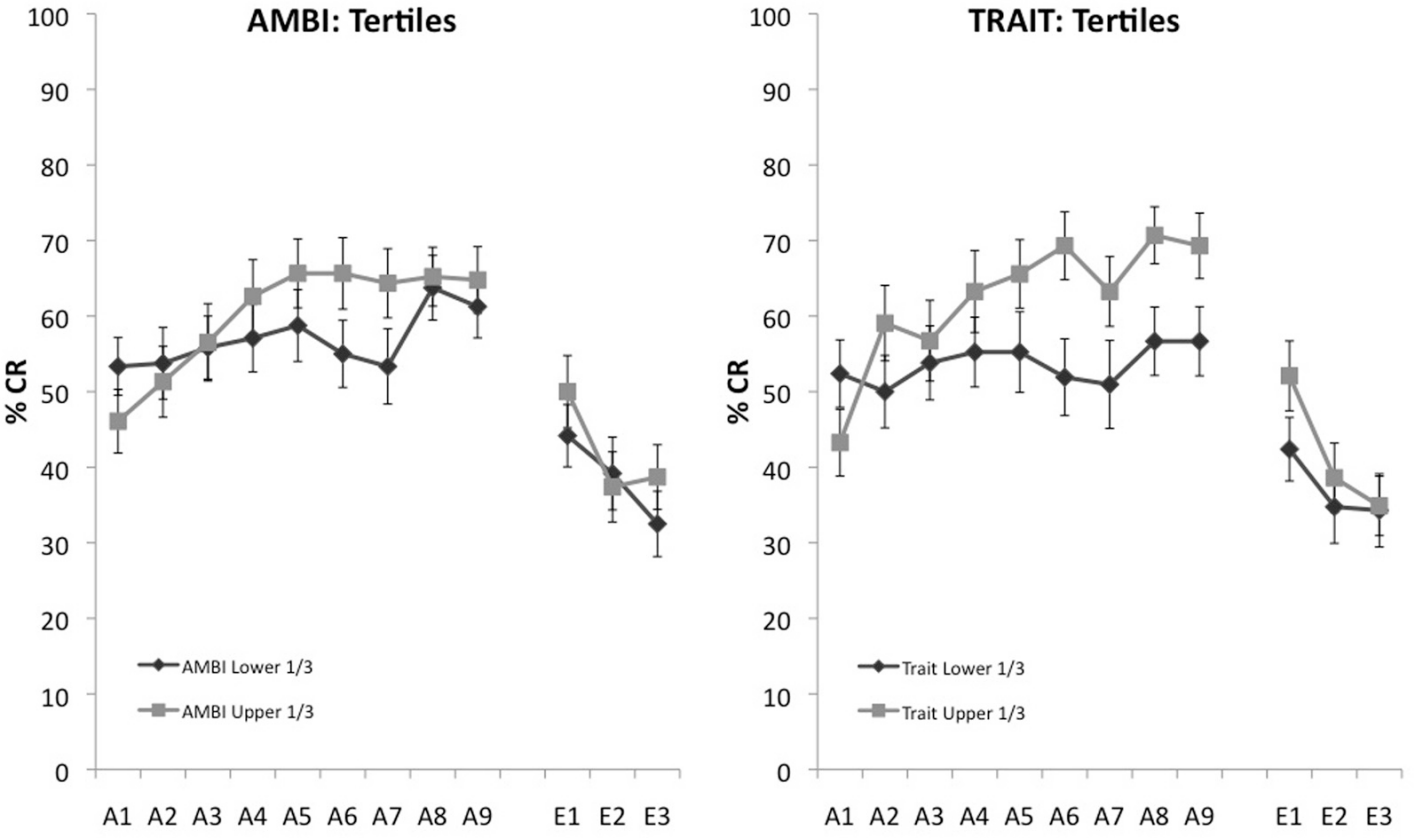
**Eyeblink conditioned responding of the upper and lower 1/3 of scores on AMBI and Trait.** Significant differences of CR acquisition remain for extreme scoring groups for both the AMBI and Trait measures.

AMBI and Trait were further analyzed to ensure that scores around the median weren't driving learning differences between groups and that the extremes of the measures maintained observed acquisition differences. To assess this, we selected the upper and lower 1/3 on the AMBI and Trait measures. A 2 (survey score: highest 1/3, lowest 1/3) × 9 (learning block) mixed measures ANOVA with UR amplitude as a covariate indicated a significant interaction, *F*_(8, 728)_ = 1.961, *p* = 0.049 between AMBI and learning. Individual differences in acquisition also remained for the highest and lowest scoring Trait groups, *F*_(8, 616)_ = 2.754, *p* = 0.005 (Figure 2).

A Spearman's rho correlation was used to further analyze the relationship between scores and the average number of CRs over the entire acquisition session of 45 trials. While AMBI was not significantly correlated with overall acquisition, *rs*[117] = 0.037, *p* = 0.689, Trait did reveal a significant positive correlation between learning and acquisition *rs*[117] = 0.186, *p* = 0.045. Furthermore, none of the other measures were significantly correlated with overall acquisition, all *p*'s > 0.247. Finally, no measures significantly correlated with extinction, all *p*'s > 0.169.

## Discussion

The present study assessed the relationship between self-report measures of anxiety vulnerability and acquisition of an associative learning task. In an extension of previous work that demonstrated faster learning in anxiety vulnerable groups, we assessed the effectiveness of measures of anxiety vulnerability to differentiate acquisition in delay-type eyeblink conditioning. We found that while highly intercorrelated, the measures did not equally discriminate between fast and slow learners.

### Anxiety vulnerability and associative learning

The present study demonstrated that individual differences in eyeblink conditioning were related to measures of anxiety vulnerability, such that those scoring high on certain measures, specifically the AMBI and STAI-Trait, acquired eyeblink conditioning faster than low scorers. Group differences between high and low scores were not significant for the other measures examined in this study, suggesting that AMBI and Trait measures may differ in some way from the CSRI/RSRI and RMBI that enables better prediction of associative learning. Considering that the AMBI and CSRI are both measures of behavioral inhibition, differences in the efficacy for the measures to separate fast and slow learners suggests that there may be fundamental differences in how these measures assess the construct of behavioral inhibition. Comparing the question and answer options for each scale reveals some potential differences. The more direct questioning method of the CSRI/RSRI, with its inclusion of questions about physical symptoms of anxiety and specific frequencies of events may fail to recognize individuals who are behaviorally inhibited but do not manifest overt symptoms of anxiousness.

In this study we demonstrated that those who endorse more AMBI questions acquire eyeblink conditioning faster. In a previous eyeblink conditioning study acquisition differences were observed in comparisons high vs. low RMBI, but not AMBI (Myers et al., 2011). One explanation for this inconsistency is sampling differences. The Myers et al. (2011) study used a sample of aged (*M* = 51.2 years) veterans with previous combat experience, unlike our sample of younger (*M* = 20.5 years) college undergraduates. Presumably, these two groups differ in many ways such as personality, motivation, and previous experience that may be reflected in self-report measures.

Another explanation for the effect of AMBI in the present study and RMBI in Myers et al. (2011) may be related to a “Do not remember” option that was available to participants in the Myers et al. (2011) study. In this case, participants who used this option received pro-rated scores based on answers to other questions on the subscale. It is possible the forced-choice nature of responses in the present study lead participants to reply inappropriately if they did not remember. Demand characteristics and accurate personal historical recall may present other possible explanations for the variations of the present study from Myers et al. (2011). Even though the study had no clinical bearing, the hospital setting in the Myers et al. (2011) study may distort answers to questions in the present compared to the past. A comparison of mean scores in the two samples reveals that veteran's RMBI scores differed by 4.7 points (from 12.6 on RMBI to 17.2 on AMBI) whereas college students differed only by 0.5 points (from 11.8 on RMBI to 12.3 on AMBI), suggesting that veterans responses are less stable between past recall and present. Together, Gladstone and Parker's (2005) BI measures have demonstrated group differences in eyeblink conditioning indicating potential for the AMBI and RMBI to differentiate associative learning. It is difficult to make direct comparisons at present given the few studies and disparate samples. Therefore, the specific role of the AMBI and RMBI remains unclear. While neither alone is the best solution, a combined solution does reveal that those scoring high on combined AMBI and RMBI have significantly more CRs overall and learn faster. Future research will assess both scales and utilize the best questions from each to capture the behaviors underlying enhanced associative learning.

Scores on the STAI-Trait also differentiated individual's associative learning. Similar to Spence and colleagues (Farber and Spence, 1953; Spence and Beecroft, 1954), participants who scored higher on the Trait measure acquired eyeblink conditioning faster and demonstrated more CRs overall than individuals scoring in the lower median, a difference that remained when comparing the upper and lower 1/3 of scores. This suggests that the MAS and STAI-Trait are measuring similar underlying constructs, although the Trait does it with a shorter form and allows comparisons to be made between a stable, long-term temperament and feelings due to a temporary state of anxiousness. This outcome is supported by Holloway et al.'s (2012) recent report of facilitated delay eyeblink acquisition following context preconditioning of those scoring high on the STAI-Trait. As a further step to ensure increased sensitivity to the US (due to state anxiety) was not sufficient to explain differences observed in acquisition we compared the magnitude of the UR and found no significant differences, indicating that increased anxiousness is not responsible for observed differences in eyeblink acquisition.

In this study, associative learning significantly correlated with Trait but not AMBI measures. Learning in eyeblink conditioning is a non-linear and dynamic process that is not the same for all individuals. For this reason, it is difficult to represent eyeblink conditioning with a single value. Overall, those with high scores on AMBI and Trait learn faster, but on an individual basis this may be due to fast learning in the first block, or a maintained high percent of CRs later, making it impossible to represent learning with a single value such as overall acquisition. Additionally, the measures used here measure the presence of anxiety vulnerability, and not its absence. Thus, a participant can only be described as higher or lower in terms of the presence of behaviorally inhibited behaviors, not if they are behaviorally “uninhibited”, thereby skewing the relationship between low scores and learning. The positive correlations for both measures with overall acquisition suggest that a larger sample or the use of a sample with extreme high and low scores may reveal the relationship between learning and AMBI.

### Risk for anxiety disorders

Individuals can be at-risk to develop anxiety disorders through a variety of vulnerabilities. A diathesis approach stresses the interaction between environment and pre-existing risk factors that increase the likelihood of developing anxiety disorders such as post-traumatic stress disorder (Mineka and Zinbarg, 2006). In addition to temperament (Spielberger et al., 1983, 1970; Rosenbaum et al., 1991; Fox et al., 2005), other risk factors include brain abnormalities (Levitt et al., 2006), genetic polymorphisms (Binder et al., 2008; Amstadter et al., 2009), previous stressful experiences (North and Smith, 1990; Davidson, 2000; Seng et al., 2009) and sex (Tolin and Foa, 2006). Individuals at increased risk for anxiety disorders process the contingencies surrounding events in their environment differently, the outcome of which is increased avoidance—a core feature of anxiety disorders (American Psychiatric Association, 2000). Here, we extend this approach to suggest the inclusion of aberrant associative learning.

### Limitations and conclusions

Females have also been found to be at greater risk than males for developing anxiety disorders. Furthermore, females demonstrate enhanced acquisition in eyeblink conditioning at times (Spence and Spence, 1966). Even though the sampling of males and females was skewed with over 2/3 female, the present study did not find a significant effect of sex in eyeblink acquisition. Additionally, sex was not significantly correlated with any of the measures of anxiety vulnerability, indicating that sex differences in risk for anxiety is not measurable by the self-report measures used in this study.

The present study was designed to use the standard approach of delay eyeblink conditioning with 100% reinforced trials in acquisition. This design was selected because it is considered the optimal parameters for CR acquisition. An important question for future research will be to understand how schedules of reinforcement and CS duration influence acquisition in anxiety vulnerable individuals.

While self-report benefits from its direct collection of individual's responses, it suffers a few drawbacks that should be acknowledged. A fundamental issue with all studies using self-report survey measures is its reliability and accuracy. It remains a concern that individuals are not as capable of honestly reporting their behaviors as desired. Retrospective recall is susceptible to forgetting, displacement, distortion. It is possible an individual with behaviorally inhibited tendencies as an adult would conform to that pattern and report similar tendencies as a child. However, strong correlations between the BI measures suggest resistance to distortion in the present study, but are unable to account for different acquisition patterns between AMBI and RMBI or with the CSRI and RSRI. Future research will have to assess how biases in self-report may relate to associative learning.

Our reliance of self-report measures of anxiety vulnerability also makes the assumption that participants are demonstrating a vulnerability to anxiety disorders in their responses, and not the preclinical manifestation of anxiety disorders. For various reasons including availability and time, this study did not use a structured clinical interview to ascertain if participants are presenting with symptoms congruent with a diagnoses of anxiety disorders. Therefore, some participants may have as-yet undiagnosed anxiety disorders. Future studies could assess the differences between anxiety vulnerability and diagnosed anxiety disorders on associative learning tasks.

This study suggests that associative learning can be differentiated with self-report scales. Here, we demonstrate that the AMBI and Trait are able to separate individuals into faster and slower learning groups. Furthermore, when the criterion was extended to the highest and lowest scoring individuals, AMBI and Trait were able to maintain significant differences between the two groups. It should be noted that the median score of 11 on both the AMBI and RMBI are very low compared to other studies, with medians reported at 16.5 and 13.5 (Gladstone and Parker, 2005), and 14 and 15 in our own studies using New Jersey college students (unpublished observations). These differences may reflect something basic about the way Midwestern students answer questionnaires, about differences in behavior or experiences as asked by the AMBI and RMBI, or about the presence of BI in the sample. Considering the inconsistency observed between the AMBI and RMBI, future studies would benefit from the use of other samples to assess the reliability and generalizability of these findings and provide a clearer understanding of the dynamic between the AMBI and RMBI measures.

The relationship between measures of anxiety vulnerability and associative learning is important to understand how risk translates to clinical anxiety. As with the vulnerabilities outlined by a diathesis model, facilitated associative learning and temperament (as measured by Trait or AMBI) may be a pre-existing risk factor that provides a pathway to developing anxiety disorders. A consistent and reliable self-report measure of anxiety vulnerability that reflects associative learning would reveal the behaviors, temperament, and underlying constructs responsible for translating risk to diagnosis in anxiety disorders.

### Conflict of interest statement

The authors declare that the research was conducted in the absence of any commercial or financial relationships that could be construed as a potential conflict of interest.

## Footnotes

^1^A 33% loss of data in this eyeblink conditioning paradigm is within the normal range (Myers et al., 2011; Holloway et al., 2012).

^2^Median splits were calculated using the upper real limit of the median score of the sample. For example, if the median is 11, the upper real limit is 11.5, with those greater classified as “high” and those scoring less classified as “low”.
